# Proteomic Exploration of Plasma Exosomes and Other Small Extracellular Vesicles in Pediatric Hodgkin Lymphoma: A Potential Source of Biomarkers for Relapse Occurrence

**DOI:** 10.3390/diagnostics11060917

**Published:** 2021-05-21

**Authors:** Ombretta Repetto, Federica Lovisa, Caterina Elia, Daniel Enderle, Filippo Romanato, Salvatore Buffardi, Alessandra Sala, Marta Pillon, Agostino Steffan, Roberta Burnelli, Lara Mussolin, Maurizio Mascarin, Valli De Re

**Affiliations:** 1 Facility of Bio-Proteomics, Immunopathology and Cancer Biomarkers, Centro di Riferimento Oncologico di Aviano (CRO), IRCCS, 33081 Aviano, Italy; asteffan@cro.it; 2 Maternal and Child Health Department, Padova University, 35128 Padova, Italy; federica.lovisa@unipd.it (F.L.); lara.mussolin@unipd.it (L.M.); 3 Istituto di Ricerca Pediatrica Città della Speranza, 35127 Padova, Italy; 4 Pediatric Radiotherapy Unit, Centro di Riferimento Oncologico di Aviano (CRO), IRCCS, 33081 Aviano, Italy; eliacaterina@libero.it (C.E.); mascarin@cro.it (M.M.); 5 Exosome Diagnostics GmbH, 82152 Martinsried, Germany; daniel@exosomedx.com; 6 Department of Physics and Astronomy, University of Padua, via Marzolo 8, 35131 Padova, Italy; filippo.romanato@unipd.it; 7 Department of Paediatric Haemato-Oncology, Santobono-Pausilipon Children’s Hospital, 0131 Napoli, Italy; salvatorebuffardi@hotmail.it; 8 Department of Paediatrics, Ospedale San Gerardo, University of Milano-Bicocca, Fondazione MBBM, 20900 Monza, Italy; ale.sala@asst-monza.it; 9 Clinic of Paediatric Haemato-Oncology, Department of Maternal and Child Health, University of Padua, 35128 Padova, Italy; marta.pillon@unipd.it; 10 Pediatric Onco-Hematology Unit, Sant’Anna Hospital, 44124 Ferrara, Italy; r.burnelli@ospfe.it

**Keywords:** biological markers, DIGE, exosomes, mass spectrometry, pediatric Hodgkin lymphoma, plasma proteins, proteomics, relapse

## Abstract

Exosomes and other small extracellular vesicles (EVs) are potential sources of cancer biomarkers. Plasma-derived EVs have not yet been studied in pediatric Hodgkin lymphoma (HL), for which predictive biomarkers of relapse are greatly needed. In this two-part proteomic study, we used two-dimensional difference gel electrophoresis (2D-DIGE) followed by liquid chromatography–tandem mass spectrometry (LC–MS/MS) to analyze EV proteins of plasma collected at diagnosis from children with nodular sclerosis HL, relapsed or not. EVs isolated using membrane affinity had radii ranging from 20 to 130 nm and contained the programmed cell death 6-interacting (ALIX) and the tumor susceptibility gene 101 (TSG101) proteins, whereas calnexin (CANX) was not detected. 2D-DIGE identified 16 spots as differentially abundant between non-relapsed and relapsed HL (|fold change| ≥ 1.5, *p* < 0.05). LC–MS/MS identified these spots as 11 unique proteins, including five more abundant in non-relapsed HL (e.g., complement C4b, C4B; fibrinogen γ chain, FGG) and six more abundant in relapsed HL (e.g., transthyretin, TTR). Shotgun LC–MS/MS on pooled EV proteins from non-relapsed HL identified 161 proteins, including 127 already identified in human exosomes (ExoCarta data). This EV cargo included 89 proteins not yet identified in exosomes from healthy plasma. Functional interrogation by the Database for Annotation, Visualization and Integrated Discovery (DAVID) revealed that the EV proteins participate in platelet degranulation and serine-type endopeptidase activity as the most significant Gene Ontology (GO) biological process and molecular function (*p* < 0.01).

## 1. Introduction

Hodgkin lymphoma (HL) is a malignant lymphoma accounting for around 5% of childhood (ages 0–14) and 15% adolescent (ages 15–19) cancers [1,2]. It is considered as a curable pediatric cancer, since most patients are cured with first-line treatment and the 5-year survival rate is 90–95% [3]. Relapses occur in about 10–20% of patients, depending on disease stage [4,5]. Great attention is currently given to these subgroups of pediatric HL patients at risk of relapse, and several studies have reported candidate biomarkers predictive of relapse [6,7,8,9].

In biomarker discovery, there is growing interest in extracellular vesicles (EVs) as a biological material that can be sampled by minimally invasive liquid biopsy to provide useful information for disease diagnosis, prognosis, and therapy [10,11]. There are three types of EVs that differ in size and biogenesis: microvesicles (diameter, 100–1000 nm) and apoptotic bodies (1–4 μm) result from plasma membrane budding [12,13], while exosomes (30–150 nm) are EVs deriving from endosomes [14]. Exosomes carry heat shock proteins (Hsp) (e.g., Hsp27, Hsp20) [15], membrane transport and fusion proteins (e.g., Ras analog in brain guanosine triphosphatases (Rab GTPases), annexins, flotillins), proteins involved in sorting of cargo into exosomes (e.g., programmed cell death 6-interacting protein, ALIX; tumor susceptibility gene 101 protein, TSG101) [16], some cell-specific proteins (e.g., major histocompatibility complex (MHC) class II and cluster of differentiation 86 (CD86)on exosomes from mature dendritic cells [17]), lipids [18], and mRNAs and non-coding RNAs [19]. They are produced by almost all kind of cells, including hematopoietic cells (B and T lymphocytes [20,21,22], platelets [23], natural-killer cells [24] and non-hematopoietic cells (intestinal epithelial cells [25], adipocytes [26], fibroblasts [27]).

Exosomes are secreted into extracellular spaces and can be internalized by recipient cells, facilitating intercellular communication and compound exchange and contributing to various processes (antigen presentation, angiogenesis, coagulation, senescence, proliferation [28]). They can also be released into biological fluids, including blood [29]. Exosome cargo reflects intracellular changes occurring under both physiological and pathological conditions, including cancer [30].

Tumor-derived exosomes may transport tumor-associated molecules, such as mRNAs, microRNAs, and proteins [31,32], and therefore may contribute to malignancy-related events (e.g., angiogenesis, tumorigenesis, tumor epithelial–mesenchymal transition, and immune escape [33,34,35]). They transport messages within stroma and distant sites by bodily fluids such as blood [36]. Therefore, exosomes isolated from bodily fluids may contain diagnostic and prognostic tumor biomarkers that can be studied in a non-invasive manner. The microRNA content of plasma-derived exosomes has been studied to monitor responses of classic HL patients to therapy [37] and to assess the impact on HL prognosis in HIV-infected HL patients [38].

During the last decade, high-throughput proteomics coupled with mass spectrometry (MS) and integrated with advanced platforms (e.g., array- or three-dimensional liquid chromatography-based, microfluidic) have offered powerful tools to finely characterize proteins in biological systems [39,40,41,42,43]. Proteomics and subproteomics methods have brought new insights to the discovery of biomarkers commonly present at low concentration levels that can be associated with disease progression [44,45]. MS-based proteomic analysis has recently been applied to cancer cell-derived EVs, revealing important biogenesis pathways and improving our understanding of carcinogenesis and tumor progression [15,46,47]. Proteomic analyses of plasma-derived exosomes have already identified potential tumor biomarkers for diagnosis (e.g., malignant pulmonary nodules [48], breast [49]) or prognosis (ovarian [50]). Thus far, a proteomic analysis of plasma-derived EVs from HL patients has not been reported.

In this comparative, quantitative proteomic study, we used two-dimensional difference gel electrophoresis (2D-DIGE) to identify proteins in plasma-derived EVs as potential biomarkers of relapse occurrence in pediatric HL. We also profiled the protein cargo of plasma-derived EVs from non-relapsed pediatric HL patients for comparison with reported protein profiles from healthy donors.

## 2. Materials and Methods

### 2.1. Research Ethics Statement

This study used plasma from patients enrolled in the multicenter “LH2004” clinical trial conducted by A.I.E.O.P. (Associazione Italiana di Emato-Oncologia Pediatrica, Bologna, Italia) for the treatment of pediatric HL and was performed in Italy from June 2004 to June 2017. The LH2004 protocol was approved by the Ethics Committee of the promoter center Azienda Ospedaliera Policlinico S. Orsola Malpighi, Bologna (Italy), (23 April 2004)(Document N° 1103/2004), and by the Ethics Committee or the Institutional Review Board of each participating institutions. Written informed consent was obtained from all participants.

### 2.2. Patients and Plasma Samples

The plasma used in this study was from 15 pediatric HL patients enrolled in LH2004 [51]. Patient inclusion criteria were having had either a favorable (non-relapsed, *n* = 9) or unfavorable (relapsed, *n* = 6) response to treatment over 5 years in the trial, being aged between 11 and 17 years, and having nodular sclerosis histology (according to [52]). Clinical data collected included stage (according to [53]), absence, or presence of systemic symptoms [53] during the 5-year follow-up and LH2004 therapeutic group. Relapse was defined as the pathologically confirmed recurrence of HL. The main clinical and biological characteristics of the study cohort are reported in Table 1.

Plasma had been obtained from blood samples collected in sodium citrate vials at HL diagnosis and aliquoted at −80 °C until analysis.

### 2.3. Study Design

Non-relapsed and relapsed HL patients were divided into two sets, the explorative and the validation sets (Table 1). An exosome-enriched fraction comprising exosomes and other small EVs (here abbreviated as EV) was isolated from plasma of both sets. In Part I, protein was extracted from the EV-enriched fraction of non-relapsed (*n* = 6) and relapsed (*n* = 3) HL patients of the explorative set and solubilized in a 2D-compatible buffer (without ionic detergent that can strongly interfere with isoelectrofocusing, IEF), and used to identify differentially abundant proteins that distinguish non-relapsed from relapsed pediatric HL. The differential pattern of some differential proteins of interest was validated by immunoblotting on pooled plasma EV proteins extracted from non-relapsed (*n* = 3) and relapsed (*n* = 3) HL patients (validation set). In Part II, protein was extracted from the EV-enriched fraction (validation set) in a buffer containing sodium dodecyl sulfate (SDS) for maximal recovery of membrane proteins and used to characterize plasma-derived EV protein cargo in non-relapsed pediatric HL (*n* = 3). Finally, the list of identified proteins was examined for prior annotation in the ExoCarta database and compared with reported protein profiles from healthy donors.

### 2.4. Isolation of EVs

Plasma samples (500 µL) were filtered to exclude particles larger than 0.22 µm (Millex-GS, Merck Millipore, Burlington, MA, USA) and processed on membrane affinity spin columns (exoEasy Midi kits, Qiagen, Germany). EVs were eluted in 300 µL buffer XE (an aqueous buffer containing primarily inorganic salts). To verify the isolation of EVs, we adsorbed a representative sample to freshly cleaved mica sheets (Agar Scientific, Stansted, UK) that was rinsed with de-ionized water, dried under a gentle stream of nitrogen, and morphologically inspected under a CP-II atomic force microscope (Veeco, Plainview, NY, USA). Measurements were performed in non-contact mode at 170 kHz resonance frequency.

### 2.5. Identification of Differentially Abundant Proteins

#### 2.5.1. Protein Extraction

**Figure 1:**
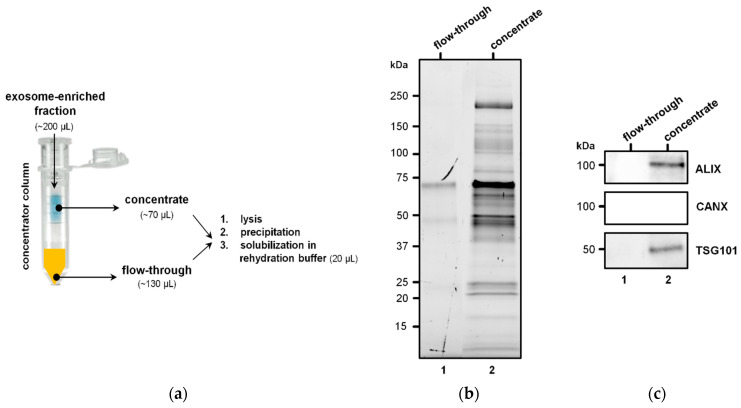
Details of the experimental approach to extract protein from EV-enriched fractions and exclude contamination of non-EV proteins. (**a**) EV-enriched fractions were concentrated with 100,000 molecular weight cut-off concentrators, and both the flow-throughs and concentrates were subjected to lysis and then purification by precipitation and solubilization in rehydration buffer (around 20 μL). (**b**) Image of a stain-free gel before protein transfer to nitrocellulose membrane: proteins from flow-throughs (10 μL) and concentrates (10 μL corresponding to 10 μg) were separated by SDS-PAGE. (**c**) Blot probed with primary antibodies against ALIX, CANX, and TSG101.

The workflow adopted to extract protein from EVs is illustrated in Figure 1a. For each sample, the exoEasy spin column eluate (“EV fraction”) containing intact vesicles was subject to protein extraction following the method of Enderle et al. [54] with some modifications.

Briefly, 200 μL of each eluate was reduced to 70 μL by centrifuging (7 min at 4000× *g*, 4 °C) in 100,000 molecular weight cut-off concentrators (Vivaspin; Sartorius, Göttingen, Germany), which retained EVs but not free proteins less than 100 kDa. Each concentrate was moved to a new tube and lysed with an equal volume of M-PER buffer (78501, Thermo Fisher Scientific, Waltham, MA, USA) containing protease inhibitors (cOmplete Tablets EDTA-free, EASYpack, Roche, Basel, Switzerland) for 1 h at 4 °C. The lysates were subjected to two cycles of freeze (−80 °C) and thaw (4 °C) and sonication (2 × 60 s). The tubes were centrifuged for 20 min at 8000× *g* to remove membrane debris, and the supernatants were processed on 2-D Clean-Up Kits (Cytiva-formerly GE Healthcare Life Sciences, Uppsala, Sweden). The resulting precipitates were solubilized in 20 μL of rehydration buffer (7 M urea (Sigma-Aldrich, St-Louis, MO, USA), 2 M thiourea (Sigma-Aldrich, St-Louis, MO, USA), 4% CHAPS (Sigma-Aldrich, St-Louis, MO, USA), 0.5% *v/v* Pharmalytes ampholyte-containing buffer (Cytiva-formerly GE Healthcare Life Sciences, Uppsala, Sweden)). Protein concentration was measured with the Pierce BCA Protein Assay Kit (Thermo Fisher Scientific, Waltham, MA, USA). Protein extracts were stored at −80 °C.

To exclude the presence of plasma protein contaminants or membrane fragments in the EV preparations, we collected the flow-throughs of the concentrators and subjected them to lysis, protein precipitation, and resuspension in 20 μL of rehydration buffer, as described for concentrates.

**Figure 2:**
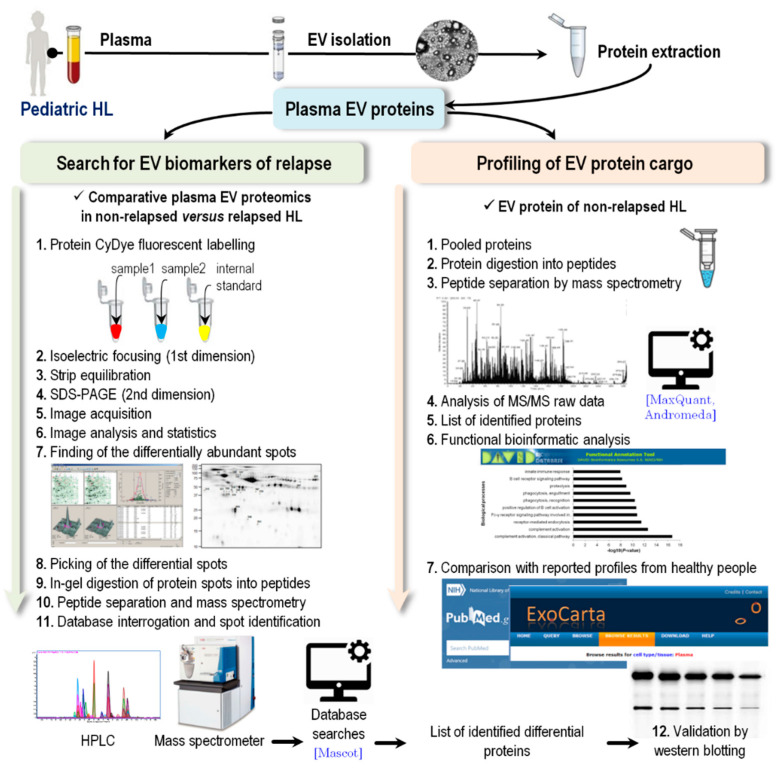
Schematic illustration of the experimental workflow adopted from plasma sampling to LC–MS/MS and computational analyses.

The experimental workflow adopted from plasma sampling is schematically illustrated in Figure 2.

#### 2.5.2. SDS-PAGE and Immunoblotting

Protein extracts were separated on 12% Criterion TGX Stain-Free gels (Bio-Rad, Hercules, CA, USA). Gel images were acquired using the Chemidoc system (Bio-Rad, Hercules, CA, USA) before transfer to nitrocellulose membranes (Protran, Whatman, Maidstone, UK). Membranes were blocked for 1 h at room temperature with 5% nonfat dry milk (Bio-Rad, Hercules, CA, USA) in Tris-buffered saline containing 0.1% Tween-20 (TBST), washed with TBST, and incubated overnight at 4 °C with primary antibodies. Membranes were washed three times with TBST, incubated with HRP-conjugated goat anti-mouse IgG (Bethyl, Montgomery, TX, USA, 1:10,000 dilution) for 1 h at room temperature, and washed five times and detected by enhanced chemiluminescence (Clarity Western EC, Bio-Rad, Hercules, CA, USA) on the Chemidoc system. Both primary and secondary antibodies were prepared in 5% bovine serum albumin in TBST.

For the validation of exosome-enrichment in the EV fractions, we analyzed protein extracts of some Vivaspin-concentrated EV fractions (10 µg in 10 µL) and flow-throughs (10 µL), and the primary antibodies were as follows: the mouse anti-TSG101 [4A10] monoclonal IgG (1:100; #ab83, Abcam, Cambridge UK); the mouse anti-human ALIX [3A9] monoclonal IgG (1:1000; #ab117600, AbCam, Cambridge UK) and the rabbit anti-human flotillin-1 (D2V7J) monoclonal IgG (1:1000; #18634, Cell Signalling Technology, Danvers, Massachusetts, USA); positive controls for exosome enrichment [55]; and the rabbit anti-human calnexin (CANX) monoclonal IgG (1:1000; #2679, Cell Signalling Technology, Danvers, MA, USA), an endoplasmic-reticulum marker and negative control for exosome isolation [55].

For the validation of selected differentially abundant proteins, we pooled EV protein extracts of three non-relapsed HL and three relapsed HL patients (validation set) (15 μg per sample). The primary antibodies were as follows: the rabbit anti-complement C4b (C4B) [3A9] polyclonal IgG (1:1000; #ab66791, AbCam, Cambridge UK), the rabbit anti-fibrinogen γ chain [EPSISR41] (FGG) monoclonal IgG (1:1000; #ab171748, Abcam, Cambridge UK), the rabbit anti-clusterin (CLU) polyclonal IgG (1:1000; #18634, Cell Signalling Technology, Danvers, MA, USA), and the mouse anti-transthyretin (TTR) polyclonal IgG (1:1000; #PA5-27220, Thermo Fisher Scientific, Waltham, MA, USA).

#### 2.5.3. Difference Gel Electrophoresis

The entire 2D-DIGE project consisted of six analytical gels, with each gel being used to analyze two protein extracts and an internal standard prepared by pooling equal amounts of all samples. Protein extracts of patients belonging to the explorative group were individually analyzed. Briefly, the protein extracts were labeled with Cy3 (*n* = 6) or Cy5 (*n* = 6), and the internal standard was labeled with Cy2 cyanine dye (CyDye DIGE Fluor minimal dyes; Cytiva-formerly GE Healthcare Life Sciences, Uppsala, Sweden). Then, two differently labeled protein extracts (25 µg each) and a similar amount of internal standard were combined and fractionated by isoelectrofocusing (IEF) on Immobiline Drystrip gels (pH 3–10 NL IPG, Cytiva-formerly GE Healthcare Life Sciences, Uppsala, Sweden) for 30 kV h on a Protean IEF Cell system (Bio-Rad, Hercules, CA, USA). The IPG strips were incubated 15 min in equilibrating buffer (4 M urea, 2 M thiourea, 50 mM Tris HCl (pH 8.9), 30% glycerol, 2% (*w/v*) SDS) containing 65 mM DTT, and 15 min in equilibrating buffer containing 135 mM iodoacetamide, and then loaded on 8–16% gradient Criterion TGX precast gels (Bio-Rad, Hercules, CA, USA) for the second dimension of electrophoresis. Reagents were from Sigma-Aldrich, St-Louis, MO, USA.

Gels were imaged using an Amersham Typhoon scanner (Cytiva-formerly GE Healthcare Life Sciences, Uppsala, Sweden; at 100 µm), and images were analyzed with DeCyder software (version 6.5; Cytiva-formerly GE Healthcare Life Sciences, Uppsala, Sweden) using the Batch Processor module, which runs the Differential In-gel Analysis (DIA) and the Biological Variation Analysis (BVA) modules automatically. In the DIA module (intra-gel analysis), spots were detected, and their staining intensity was normalized to that of the same spot of the internal standard (standardized abundance). In the BVA module (inter-gel analysis), the standardized abundance of spots across gels was compared, and the average ratio (fold change) between the relapsed and non-relapsed groups was calculated. Differentially abundant spots between relapsed and non-relapsed groups were those that had an average ratio (fold change) ≥1.5 or ≤−1.5 and had *p* < 0.05 on Student’s *t*-test. The Extended Data Analysis module of DyCyder was used to run principal component analysis: differentially abundant spots were analyzed in a score plot, and the patients’ spot maps were analyzed in a loading plot.

An a priori power analysis was conducted to estimate the minimum detectable effect between 6 non-relapsed and 3 relapsed HL patients. Fixing α = 0.05, β = 0.20, the minimum fold change was 1.5 when the covariance was 0.20, according to a two-sided *t*-test.

Differentially abundant spots were excised from a 2D-DIGE Coomassie blue-stained preparative picking gel (200 µg of unlabelled proteins coming from all 9 patients of the explorative set) with Screen Picker (Proteomics Consult). Excised spots were destained overnight with 25 mM ammonium bicarbonate in 50% acetonitrile (both from Sigma-Aldrich, St-Louis, MO, USA), and then dehydrated with 100% acetonitrile and dried. Next, they were digested overnight with trypsin (200 ng/spot; T6567 Sigma-Aldrich, St-Louis, MO, USA) in 40 mM ammonium bicarbonate and 9% acetonitrile at 37 °C. The tryptic digest was then extracted with 1% trifluoroacetic acid (Sigma-Aldrich, St-Louis, MO, USA), lyophilized, and stored at −80 °C until shipping.

#### 2.5.4. Protein Identification by Mass Spectrometry

Samples were shipped at −80 °C to the Proteomics Facility of CEINGE-Biotecnologie Avanzate (Naples, Italy) for LC–MS/MS, which was performed on a Proxeon EASY nano liquid chromatography system coupled with an LTQ Orbitrap XL mass spectrometer (Thermo Fisher Scientific, Waltham, MA, USA).

Searches for matches between the MS data and proteins in the NCBI nr and Swiss-Prot databases (human taxonomy) were performed with the Mascot Server v2.3 (Matrix Science, Boston, MA, USA). Searches were conducted with carbamidomethylation of cysteine as a fixed modification; oxidation of methionine, pyroglutamate formation from glutamine and pyro-carbamidomethyl as variable modifications of proteins; a peptide mass tolerance of ±10 ppm; a fragment mass tolerance of ±0.6 Da; and an allowance for up to two missed tryptic cleavages.

### 2.6. EV Protein Cargo Analysis in Non-Relapsed Pediatric HL

#### 2.6.1. Protein Extraction

Protein was extracted from EVs essentially as described in Section 2.5.1, with the exception that the M-PER lysis buffer was supplemented with 3% SDS and protein concentrates were not processed on 2-D Clean-Up kits. Protein was quantified with the Pierce BCA Protein Assay Kit (Thermo Fisher Scientific, Waltham, MA, USA). Protein extracts were stored at −80 °C. Protein extracts of three non-relapsed HL patients (explorative set) were pooled (25 μg per sample) and digested with trypsin in S-Trap spin columns (Protifi, Farmingdale, NY, USA) according to the manufacturer’s procedure as recently reported [6].

#### 2.6.2. Protein Identification by Shotgun LC–MS/MS

The peptide digest was solubilized in formic acid and analyzed in duplicate (technical replicates) by LC–MS/MS by the Facility of Proteomics of CEINGE-Biotecnologie Avanzate (Naples, Italy) using an LTQ Orbitrap XL mass spectrometer (Thermo Fisher Scientific, Waltham, MA, USA). After loading, the sample was concentrated and desalted on a C18 Easy-Column (L = 2 cm, ID = 100 μm; cat. no. 03-052-619, Thermo Fisher Scientific SC001, Waltham, MA, USA). Then, it was separated on a C18 reverse-phase capillary column (L = 20 cm, ID = 7.5 μm; cat. no. NS-AC-12, Nano Separation, Niewkoop, the Netherlands) at a flow rate of 250 nL/min in a gradient from 5% to 95% buffer B (eluent B: 0.2% formic acid in 95% acetonitrile, Sigma-Aldrich (St-Louis, MO, USA); eluent A: 0.2% formic acid and 2% acetonitrile in ultrapure water) over 285 min.

The mass spectrometer was operated in positive polarity mode with capillary temperature of 275 °C. MS/MS analyses were performed using Data-Dependent Acquisition (DDA) mode: one MS scan (mass range from 400 to 1800 *m/z*) was followed by MS/MS scans of the 10 most abundant ions in each MS scan, applying a dynamic exclusion window of 40 s. The sample was run in technical duplicates. The LC–MS/MS raw data were analyzed with MaxQuant 1.6.17.0 (RRID:SCR_014485; https://www.maxquant.org/) integrated with the Andromeda search engine. The selected parameters for protein identification were as follows: carbamidomethylation of cysteine as a fixed modification; 2 max. missed cleavages; 1 min. unique peptide; 3 min. razor peptides; 3 min. peptides; protein FDR value = 0.01; protein quantification using unique and razor peptides; min. peptide length = 7; use of human FASTA-file. Spectral counts of technical duplicates were averaged.

#### 2.6.3. Protein Functional Annotation

Functional annotation of the proteins identified by shot-gun LC–MS/MS in EVs of non-relapsed pediatric HL was performed with DAVID 6.8 [56]. This method showed the involved gene ontology (GO) biological processes, molecular functions, and cellular components associated with the proteins together with *p*-values (Fisher’s exact test) and Benjamini-corrected *p*-values. Strongly enriched annotation categories (Benjamini-corrected *p* < 0.01) were considered.

### 2.7. Interrogation of ExoCarta and Comparison with Previous Research

Proteins identified by 2D-DIGE and shotgun LC–MS/MS were searched for in ExoCarta (http://exocarta.org, accessed on 8 February 2021), an online database of exosomal proteins, RNAs, and lipids [57]. On the day of analysis, the database had information about 214 proteins in plasma-derived exosomes. We also compared our findings with those of three studies of plasma exosomes of healthy individuals [58,59,60], and generated a symmetric Venn diagram (http://bioinformatics.psb.ugent.be/webtools/Venn/, accessed on 26 March 2021) to show proteins either shared by the other three works or unique to our study.

## 3. Results

### 3.1. EVs from Plasma of Pediatric HL Patients

This study used plasma from 15 children with nodular sclerosis type of HL, including 9 with non-relapsed HL and 6 with relapsed HL (Table 1). Eight patients had stage 2 disease and seven had stage 4 disease; 8 of the 15 had at least one systemic symptom.

EV preparations were concentrated in Vivaspin concentrators with a 100,000 molecular weight cut-off, and then the concentrate and flow-through were subjected to protein extraction (Figure 1a). SDS-PAGE of the flow-through revealed the absence of protein (Figure 1b, lane 1), indicating that the EV preparations were not strongly contaminated with plasma protein or membrane fragments. SDS-PAGE of the concentrated EVs showed a mixture of molecules of different sizes, but an abundance of proteins with molecular weight > 50 kDa (Figure 1b, lane 2). Immunoblotting confirmed the presence of two exosome markers, TSG101 and ALIX, and the absence of CANX, in the concentrated EV fraction (Figure 1c, lane 2).

**Figure 3:**
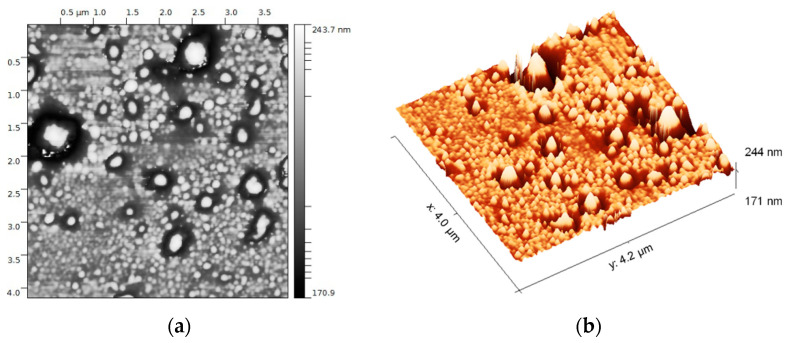

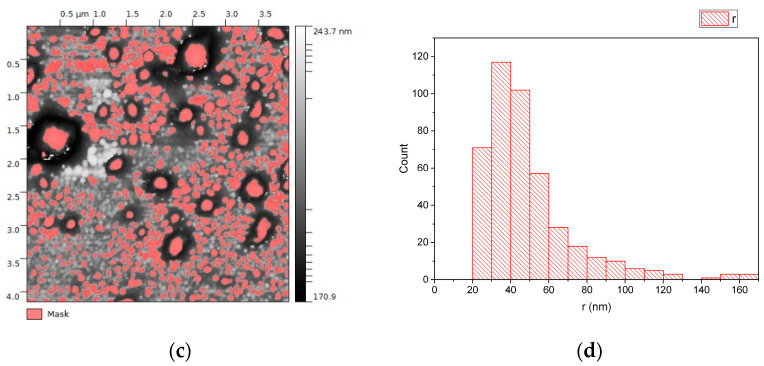
Non-contact mode (170 kHz) atomic force microscopy (AFM) of intact EVs isolated from 500 µL of plasma from a single patient with pediatric HL: (**a**) 2D AFM image of EVs diluted 1:100; (**b**) 3D AFM image of EVs diluted 1:100; (**c**) mask detection of the vesicles for statistical analysis; (**d**) radius size distribution of EVs.

EVs were extracted from plasma using a membrane affinity method that produces an exosome-enriched fraction. Atomic force microscopy of one representative sample showed roundish particles between 20 and 170 nm radius (Figure 3a,b). Size analysis revealed that >90% had a diameter in the 40–120 nm range (Figure 3c,d), consistent with the expected size distribution of an exosome-enriched sample. Since the samples were prefiltered to exclude particles > 220 nm, particles with radius > 110 nm, representing <10% of counted particles, were considered artifacts (possibly aggregates).

### 3.2. Differential Profiles of EV Proteins between Non-Relapsed and Relapsed HL

**Figure 4:**
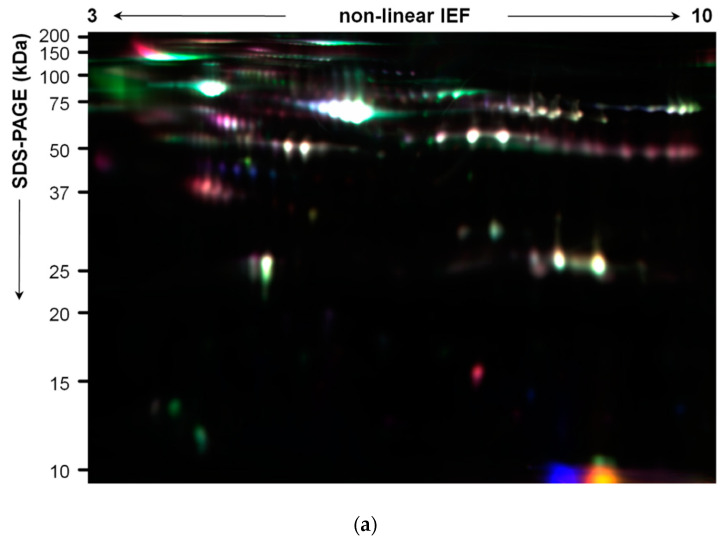

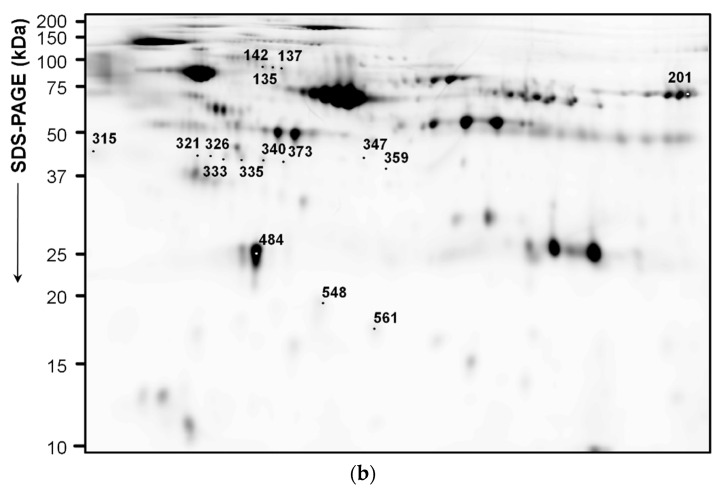
Two-dimensional proteome maps of pediatric HL EVs. Proteins were resolved by IEF on immobilized pH 3–10 non-linear gradients, followed by SDS-PAGE (12.5%). (**a**) A DIGE image of a picking gel loaded with 25 µg each of EV protein from two non-relapsed HL patients (stained with Cy3 or Cy5 dye) and an equal amount of the Cy2-labeled internal standard. (**b**) Coomassie blue-stained preparative gel loaded with 200 µg protein. The numbers indicate the differentially abundant spots between non-relapsed and relapsed HL groups (|fold change| ≥ 1.5, Student’s *t*-test *p* < 0.05).

In Part I of this study, plasma EV proteins were resolved by 2D-DIGE, generating proteome maps (Figure 4a). Comparison of proteome maps between non-relapsed and relapsed pediatric HL patients found 16 differentially abundant spots (*p* < 0.05, Student’s *t*-test) (Table 2).

Six spots were more abundant in non-relapsed HL and 10 spots were more abundant in relapsed HL (Figure 4b, numbers). They were identified by LC–MS/MS (Table 2).

In non-relapsed pediatric HL, the more abundant spots were identified as five unique proteins: isoform 2 preproprotein of complement C4-A (C4-A), complement C4-B (C4B), fibrinogen γ chain (FGG), inter-α-trypsin inhibitor heavy chain H2 (ITIH2), and immunoglobulin heavy chain constant region mu (IGHM). In relapsed pediatric HL, the more abundant spots were identified as six distinct proteins: apolipoprotein A-I (APOA1), apolipoprotein A-IV (APOA4), clusterin (CLU), haptoglobin (HP), α-1-acid glycoprotein 1 (ORM1), and transthyretin (TTR).

**Figure 5:**
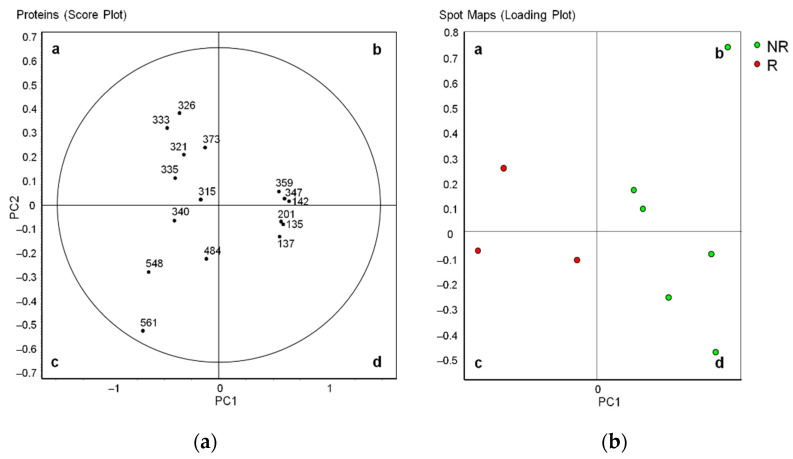
Principal component analysis of 19 differentially abundant spots. (**a**) Score plot, with each circle representing a spot (protein). The 95% significance level is marked by the circle. (**b**) Loading plot, with each circle representing a spot map of a single patient. Green dots, non-relapsed HL; red dots, relapsed HL.

Principal component analysis (PCA) showed that all 16 differentially abundant spots entered the 95% significance in the score plot (Figure 5a). In the loading plot analysis, the non-relapsed and relapsed pediatric HL groups formed two distinct clusters (Figure 5b).

### 3.3. Immunoblotting Validation of Differentially Abundant Proteins

**Figure 6:**
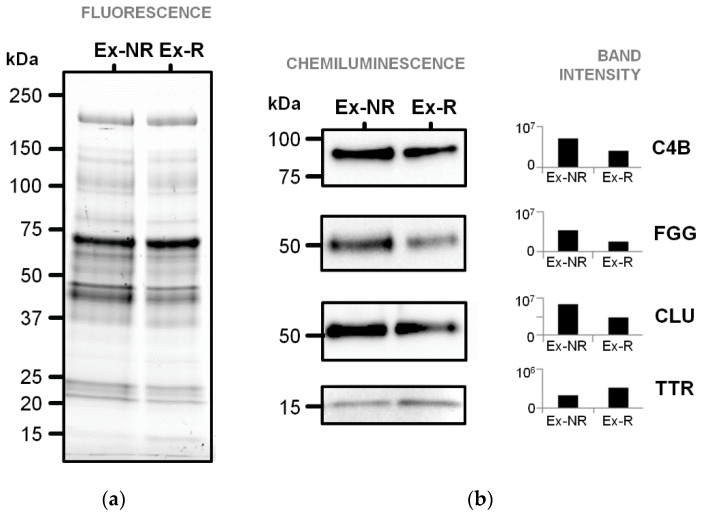
Immunoblotting detection of four differential plasma EV proteins (gene names on the right) of non-relapsed and relapsed pediatric HL patients. (**a**) Image of the stain-free 1DE gel (15 µg protein/lane) upon excitation with the Chemidoc system before its transfer to the nitrocellulose membrane. (**b**) Chemiluminescence signals of bands cross-reacting with the antibodies and their intensities calculated with the Image LabTM software. Ex-NR, exosomal proteins of non-relapsed HL; Ex-R, exosomal proteins of relapsed HL.

The abundance of four selected proteins was examined by immunoblotting of pooled plasma EV proteins (Figure 6). This analysis confirmed the higher levels of FGG and C4B in plasma EVs of non-relapsed pediatric HL and the higher level of TTR in plasma EVs of relapsed pediatric HL, but did not confirm the higher level of CLU in plasma EVs of relapsed pediatric HL.

### 3.4. EV Protein Cargo in Non-Relapsed Pediatric HL

In Part II of this study, EV protein extracts from three patients with non-relapsed HL (explorative group) were pooled and profiled by LC–MS/MS (Supplementary Table S1). This analysis identified 161 proteins within the EVs (Supplementary Table S2). ExoCarta database interrogation revealed that 128 (80%) of these proteins had already been identified in exosomes of various human sample materials, including 31 proteins in plasma-derived EVs (asterisks in Supplementary Table S2; Supplementary Table S3a). Among the 31 proteins, two (C4b-binding protein β chain and proteoglycan 4) had been identified only in exosomes from plasma and not from other materials. Finally, the other 34 proteins (e.g., SPARC-like protein 1, plasma protease C1 inhibitor, antithrombin-III, and chromogranin-A) have not yet been described in EVs according to ExoCarta.

The 161 EV proteins identified by shotgun LC–MS/MS included all 11 differentially abundant proteins found by 2D-DIGE (Supplementary Table S2, degree signs), and according to ExoCarta, six of these had already been identified in human plasma exosomes (asterisks in Table 2). These EV proteins included three more abundant in non-relapsed pediatric HL (C4B, C4A, and FGG) and three more abundant in relapsed HL (CLU, HP, and TTR). The other five differentially abundant proteins had already been identified in exosomes from other human sample materials (e.g., ITIH2, in EVs from platelets and B, melanoma, mesenchymal stem, ovarian cancer, and prostate cancer cells; ORM1, in EVs from urine) (double asterisks in Table 2).

**Figure 7:**
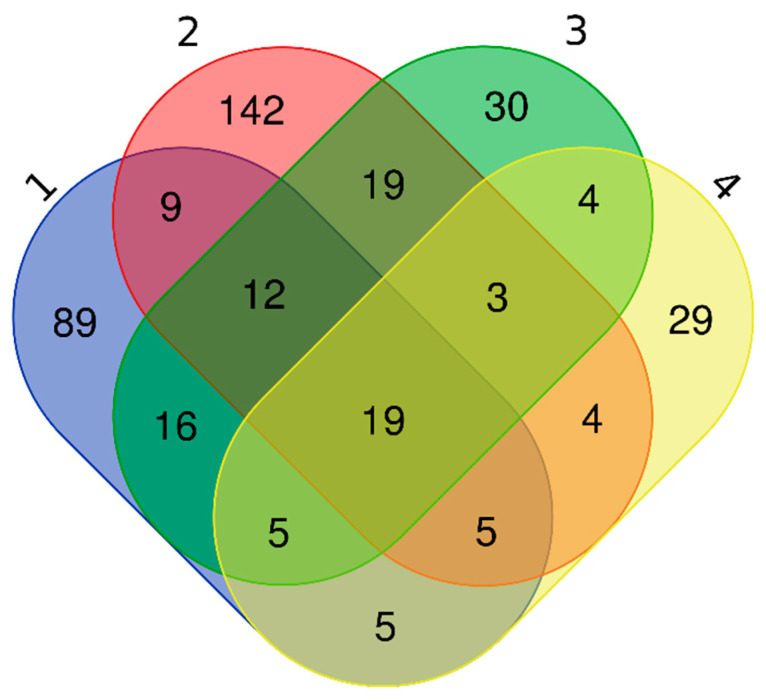
Comparison of proteins detected in our work in plasma-derived EVs from non-relapsed pediatric HL and in three works in plasma-derived EVs from healthy subjects (1: our work; 2: [58]; 3: [59]; 4: [60]). Venn diagram illustrates proteins shared by the four works and those unique of each work.

Because ExoCarta does not permit searching by disease state, we compared our list of 161 plasma-derived EV proteins of non-relapsed pediatric HL to those of three studies of plasma-derived EVs from healthy subjects (Supplementary Table S2, right columns; Figure 7). This analysis revealed that 89 proteins (55%) had not been identified in EVs of healthy plasma in any of the studies and therefore could be plasma EV proteins related to pediatric HL (Supplementary Table S3b). Only 19 of our EV proteins were common to all three other studies, including complement C4A and C4B, clusterin, fibrinogen γ chain, and haptoglobin found by DIGE (Supplementary Table S3a).

Fifty-two proteins (32%) had been identified in healthy plasma EVs by Bastos-Amador et al. [59], 34 proteins (21%) had been identified in healthy plasma EVs by Looze et al. [60], while 45 (28%) had been identified by Kalra et al. [58].

### 3.5. Functional Annotations

**Figure 8:**
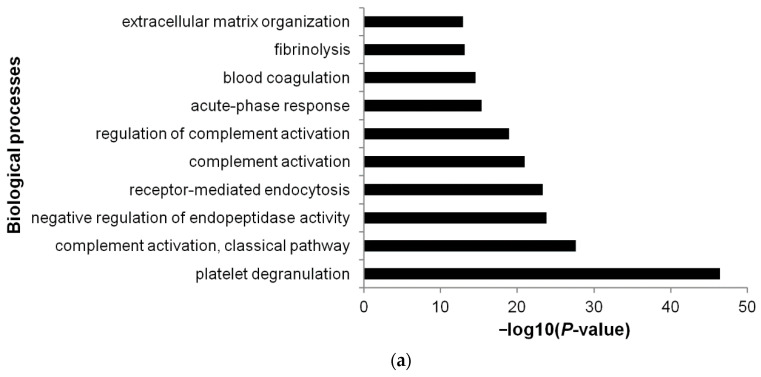

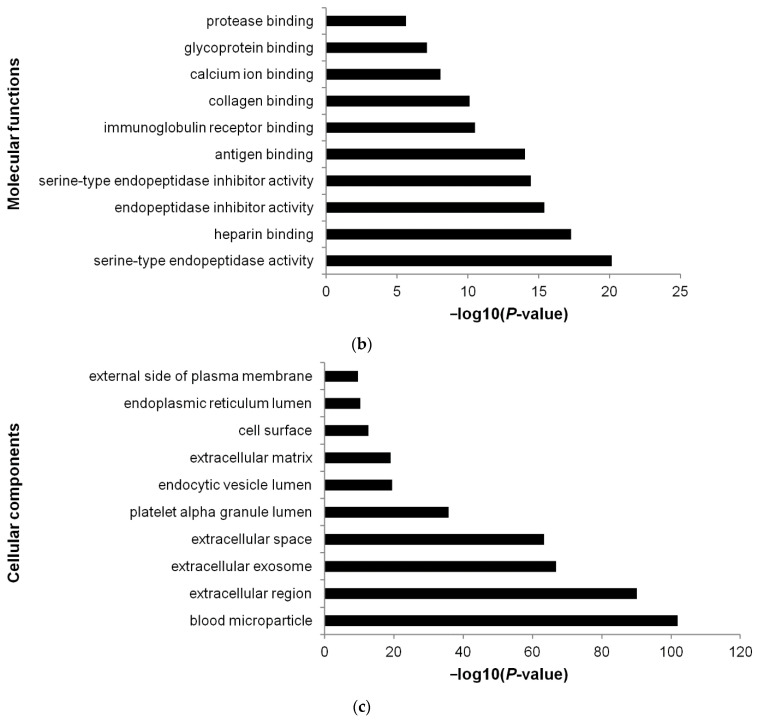
Top 10 most significant (Benjamini-corrected *p* < 0.01) biological processes (**a**), molecular functions (**b**), and cellular components (**c**) associated with plasma EV proteins of non-relapsed HL according to DAVID Bioinformatics Resources interrogated with the 161 proteins identified by LC–MS/MS (Supplementary Table S2).

DAVID Bioinformatics Resources were used to obtain GO functional annotations for the 161 EV proteins of non-relapsed pediatric HL. The most significant biological process was platelet degranulation (GO:0002576), followed by complement activation, classical pathway (GO:0006958) (Figure 8a; Supplementary Table S4a). The most significant molecular function was serine endopeptidase activity (GO:0004252), followed by heparin binding (GO:0008201) (Figure 8b; Supplementary Table S4b). The most significant cellular component was blood microparticle (GO:0072562), followed by extracellular region (GO:0005576) (Figure 8c; Supplementary Table S4c).

**Figure 9:**
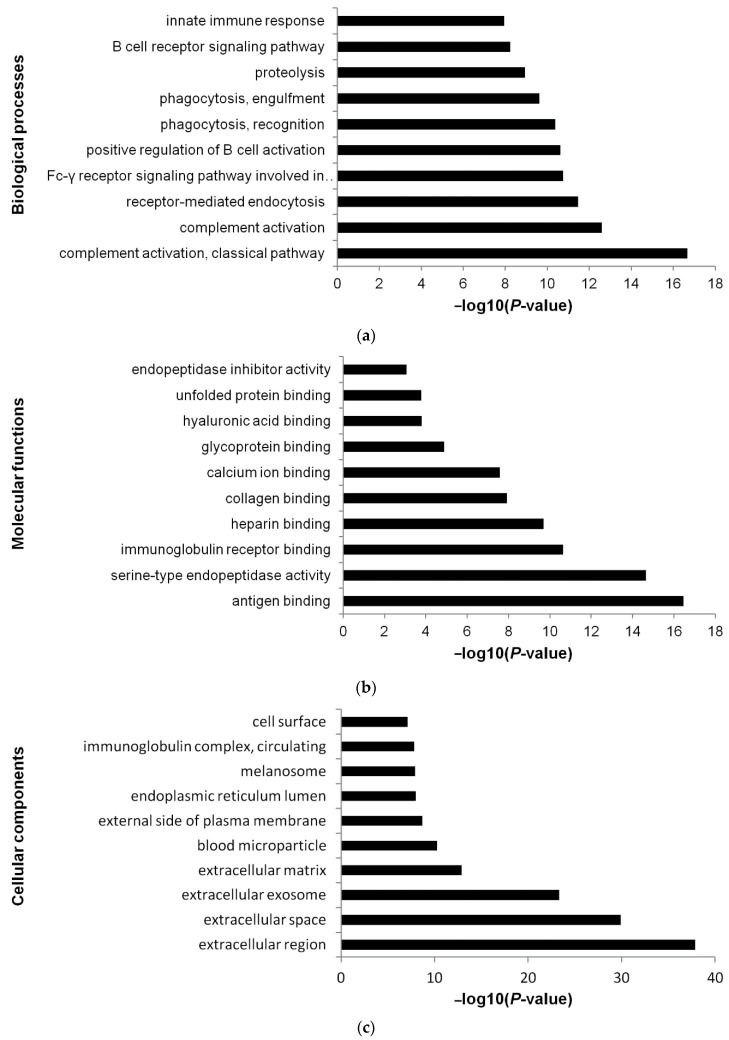
Top 10 most significant (Benjamini-corrected *p* < 0.01) biological processes (**a**), molecular functions (**b**), and cellular components (**c**) associated with plasma EV proteins of non-relapsed HL according to DAVID Bioinformatics Resources interrogated with the proteins identified LC–MS/MS after eliminating the proteins reported as detected in plasma exosomes of healthy donors (Supplementary Table S3b).

Limiting the analysis to the 89 EV proteins unique to our study (not identified in three studies of healthy plasma EVs), we found that the most significant biological process was complement activation, classical pathway (GO:0006958); the most significant molecular function was antigen binding (GO:0003823); and the most significant cellular component was extracellular region (GO:0005576) (Figure 9a–c; Supplementary Table S4d–f).

## 4. Discussion

This comparative and quantitative proteomic study based on 2D-DIGE and LC–MS/MS identified 11 plasma-derived EV proteins whose levels at diagnosis of pediatric HL associated with either the absence or the presence of relapse. Of these proteins, five were more abundant in non-relapsed HL and six were more abundant in relapsed pediatric HL. In a shotgun LC–MS/MS approach, 161 proteins were identified within plasma EVs of non-relapsed pediatric HL, and 89 of these proteins were assumed to be related to pediatric HL. According to DAVID, these 89 proteins were enriched in complement activation, classical pathway and antigen binding as the more abundant biological process and molecular function, respectively.

Exosomes and other EVs were enriched from plasma of pediatric HL patients with a membrane affinity spin column method [54]. Plasma pediatric HL EVs were spherical and mostly 40–120 nm in diameter distribution. The presence in the EV extracts of the two typical exosomal proteins TSG101 and ALIX together with the absence of the ER protein CANX confirmed the purity of the exosome-enriched fractions obtained by the isolation procedure. The method we adopted to prepare EV-rich fractions has the advantage of quickly purifying functional exosomes from a reduced plasma volume and with low-speed centrifugation. As found by [61], it suffers the limit of containing small amounts of non-EV lipid particle lipoproteins such as chylomicrons (200–600 nm diameter [62]) and very low density lipoproteins (VLDL; 30–90 nm diameter [63]). In this context, we identified apolipoprotein A-I (APOA1) as more abundant in EVs of relapsed HL, and isoforms of apolipoprotein A-IV (APOA4) among proteins more abundant in EVs of both non-relapsed and relapsed HL.

Globally, the proteome 2D map of our plasma EVs from pediatric HL patients was similar to that of plasma EVs from patients with Parkinson’s disease [63], that is, to our knowledge, the only proteome 2D map available of human plasma EVs. The number of differential spots (*n* = 16) is consistent with that one of previous works adopting DIGE to characterize differential protein profiles in exosomes isolated from human plasma [64] or serum [65].

Plasma EVs of non-relapsed pediatric HL at diagnosis possessed a high abundance of fibrinogen γ chain (FGG), and high levels of FGG were previously found in plasma of relapsed pediatric HL at diagnosis [6,7]. Similarly, in plasma EVs of relapsed HL at diagnosis, our work showed the abundance of transthyretin (TTR), and high levels of TTR were previously found at diagnosis in plasma of non-relapsed pediatric HL [6].

Both FGG and TTR are mainly synthesized and secreted into the blood by liver [66,67], with an extrahepatic synthesis also being reported for both fibrinogen (e.g., intestine and lung epithelium [68]) and TTR (e.g., intestine, pancreas [66]). In particular, FGG is a chain of a positive acute phase protein (its plasma concentration increases during inflammation) [69], while TTR is a negative acute-phase reactant [70] (its plasma concentration decreases during inflammation). FGG is a subunit of fibrinogen with a pivotal role in coagulation, inflammation [71,72], and cancerogenesis [73,74]. It has been proposed as diagnostic markers in several diseases (e.g., non-small-cell lung cancer [75], bladder cancer [76]). TTR is a carrier of thyroid hormone and retinol in plasma, and cerebrospinal fluid [77] is an important nutrition indicator, a predictive marker for liver function [78], and a biomarker of several oxidative stress-related human diseases [79].

Further investigations may help to understand the occurrence of FGG and TTR sequestration in plasma EVs of non-relapsed and relapsed pediatric HL, respectively, and their abundance in circulating EVs opposite to those observed in plasma.

Plasma EVs of non-relapsed pediatric HL at diagnosis also showed a high abundance of complement C4B, an isotype of C4 protein, that is a fundamental protein playing key roles in both the intricate complement system and the innate immunity against invading pathogens and “nonself” cells [80]. An interplay between the complement system and cancer cells occurs in the tumor microenvironment, affecting tumor progression, metastasis, and recurrence [80].

All the differentially abundant proteins found by our DIGE-based approach are major, abundant constituents of plasma. Among them, plasma exosomal FGG was recently found with fibrinogen β chain as a protein marker discriminating benign from malignant pulmonary nodules [48], being a promising prognostic biomarker for ovarian cancer [50] or cardiovascular disease [81].

The interrogation of ExoCarta with the 161 proteins identified by the shot-gun MS approach in plasma EVs of non-relapsed pediatric HL showed around 80% proteins deposited in various human sample materials, with 21% being in plasma of healthy subjects [60]. The comparison of our plasma EV protein cargo with those reported by other two works in circulating exosomes from normal human plasma [58,59] allowed us to select 19 proteins common to all the papers and 89 unique of ours. These 89 proteins were considered as potential EV markers of pediatric HL disease. Among the 19 shared proteins, some (C4A, C4B, CLU, FGG, HP) were found to be differential by 2D-DIGE, reinforcing our findings of their exosome localization.

It should be noted that these works adopted methods to isolate exosomes from plasma different for ours, and this may have affected the spectrum of proteins identified by MS analysis.

The biological process “platelet degranulation” (36 proteins; e.g., multimerin 1, MMRN1; clusterin, CLU; cathepsin G, CTSG), occurs as one of the most significant in plasma EV proteins of non-relapsed pediatric HL. Platelet activation itself can lead to the secretion of α-granules, microvesicles (100 nm–1 µm), and exosomes measuring 40 to 100 nm in diameter, enriched in the CD63 protein [23] and potentiating inflammatory responses [82]. In our plasma EV samples, we cannot attribute a cell origin of these functions associated with platelet degranulation.

The plasma circulating EVs of pediatric HL showed a high significant content of the molecular function “serine-type endopeptidase activity” (27 proteins; e.g., plasminogen, PLG; vitamin K-dependent protein Z, PROZ; vitamin K-dependent protein C, PROC). Serine-type endopeptidase (EC 3.4.21) is a large family of proteolytic enzymes (e.g., trypsin, chymotrypsin, elastase, collagenase, thrombin) [83], playing key roles in many biochemical processes (e.g., blood coagulation and fibrinolysis, apoptosis, extracellular matrix degradation and inflammation) [84,85]. The plasma exosomal cargo we observed enriched in serine proteases may provide these functions to the hypothetical recipient cells/tissues. Other exosome proteases, mostly surface-bounded, have been reported as playing critical roles in tissue remodeling and cancer progression [86].

The functional annotation based only on the 89 plasma EV proteins unique to our work showed that “complement activation, classical pathway” (25 proteins; e.g., complement C3, C3; complement C1s subcomponent, C1S; complement component C9, C9) became the most significant biological process, while “antigen binding” (16 proteins; e.g., immunoglobulin heavy constant α 1, IGHA1; HLA class I histocompatibility antigen, A-29 α chain, HLA-A; HLA class I histocompatibility antigen, Cw-7 α chain, HLA-C) was the most significant molecular function.

Recently, extracellular vesicles have been defined by Karasu et al. [87], “packages sent with complement”, with the authors emphasizing the limited current knowledge on the crosstalk between EVs and the complement system. The complement system is essential to innate immunity and an important player in inflammatory and immune responses. An exosome-induced immunity is known [88].

Plasma exosomes have been shown to carry molecules involved in immune activation (e.g., HLA-A [89]). Exosomes can express major histocompatibility complex class I and II [90], and they have been recently defined as “efficient nano-messengers of antigen presentation” [91]. Moreover, EVs released from innate immune cells can mediate innate immune responses [92,93]. In HL pathogenesis, the contribution of innate immune system has been investigated in regards to the interactions between Hodgkin and Reed-Sternberg cells and innate immune cells (e.g., macrophages, dendritic cells, natural killer cells) and their release of cytokines/chemokines (reviewed by [94]). At present, there still a lack of knowledge about the potential role of exosomes in pediatric HL disease and immunity.

## 5. Conclusions

In plasma EVs of pediatric HL patients, our study identified a differential protein display as potentially predictive of relapse: a decrease in FGG and C4B abundances together with an increase in TTR content occurred at diagnosis in relapsed HL. A putative protein cargo of plasma EVs related to pediatric HL disease was individuated as mostly enriched in proteins involved in “complement activation, classical pathway” biological process and “antigen binding” molecular function. Our proteomic approach should be integrated with other analyses deciphering the protein cargo of plasma EVs isolated from relapsed pediatric HL as well as the potential cell/tissue origin of the identified EV proteins. Moreover, the prognostic value of our differentially abundant plasma EV proteins should be tested on a larger cohort of HL patients with different prognosis.

## Figures and Tables

**Table 1:** Clinicopathological characteristics of patients with pediatric Hodgkin lymphoma (all nodular sclerosis type) who had either a favorable (non-relapsed) or unfavorable (relapsed) response to treatment in the LH2004 trial and from whom EVs were isolated.

	Explorative Set (Part I: 2D-DIGE)		Validation Set (Part I: Immunoblotting; Part II: Shotgun LC–MS/MS)	
Characteristic	Non-Relapsed HL (*n* = 6)	Relapsed HL (*n* = 3)	Non-Relapsed HL (*n* = 3)	Relapsed HL (*n* = 3)
Gender				
Male	4	3	1	6
Female	2	0	2	0
Age at diagnosis, years, mean (SD)	14 (2)	14 (3)	15 (1)	17 (1)
Stage, *n* ^a^				
II	3	2	3	0
IV	3	1	0	3
Systemic symptoms, *n*	3	2	1	2
LH2004 therapeutic group				
1	1	0	0	0
2	2	0	3	0
3	3	3	0	3

^a^ According to [53].

**Table 2:** Differentially abundant proteins in plasma-derived EVs between patients with non-relapsed and relapsed pediatric HL of the explorative groups.

Spot No. ^a^	MW (pI)	Database	Accession	Gene	Protein	Score	Seq. cov. % ^b^	Fold Change ^c^	*p*-Value
**More abundant in non-relapsed HL (*n* = 6)**									
142	106,853 (6.40)	SwissProt	ITIH2_HUMAN	ITIH2	Inter-α-trypsin inhibitor heavy chain H2 **	472	19	−2.0	0.033
135	194,170 (6.89)	SwissProt	CO4B_HUMAN	C4B	Complement C4-B *	351	6	−2.0	0.018
137	189,125 (6.70)	NCBInr	gi|356582273	C4A	Complement C4-A isoform 2 preproprotein *	337	7	−1.8	0.00632
201	189,599 (7.39)	NCBInr	gi|1314244	C4B	Complement C4B precursor	812	11	−1.8	0.00179
347	52,106 (5.37)	SwissProt	FIBG_HUMAN	FGG	Fibrinogen γ chain *	174	29	−1.6	0.033
359	50,117 (6.40)	NCBInr	gi|12054080	IGHM	Immunoglobulin heavy chain constant region mu **	148	11	−1.5	0.042
**Less abundant in non-relapsed HL (*n* = 10)**									
315	23,725 (4.93)	NCBInr	gi|112877	ORM1	α-1-Acid glycoprotein 1 **	569	33	1.6	0.049
373	15,991 (5.52)	SwissProt	TTHY_HUMAN	TTR	Transthyretin *	275	56	1.7	0.049
484	28,061 (5.27)	NCBInr	NP_001304947.1 °	APOA1	Apolipoprotein A-I isoform 1 preproprotein	7462	92	1.7	0.0062
326	30,759 (5.56)	SwissProt	APOA1_HUMAN	APOA1	Apolipoprotein A-I **	524	56	2.1	0.030
321	53,031(5.89)	SwissProt	CLUS_HUMAN	CLU	Clusterin *	209	17	2.2	0.019
340	45,371 (5.28)	SwissProt	APOA4_HUMAN	APOA4	Apolipoprotein A-IV **	294	44	2.3	0.018
335	45,371 (5.28)	NCBInr	gi|178759	APOA4	Apolipoprotein A-IV	700	61	2.3	0.014
333	45,371 (5.28)	SwissProt	APOA4_HUMAN	APOA4	Apolipoprotein A-IV	314	56	2.5	0.016
548	45,861 (6.13)	SwissProt	HPT_HUMAN	HP	Haptoglobin *	397	10	3.2	0.00895
561	45,861 (6.13)	SwissProt	HPT_HUMAN	HP	Haptoglobin *	261	10	3.3	0.017

^a^ Spot numbers refer to Figure 4; ^b^ Seq. cov. %, percentage of sequence coverage; ^c^ fold change, average ratio of the standardized spot abundance between relapsed and non-relapsed groups. ° This NCBI reference sequence replaces gi|90108664. * Previously identified in exosomes from human plasma; ** previously identified in exosomes from non-plasma human materials (www.exocarta.org, accessed on 8 February 2021).

## Data Availability

All data are included in the paper.

## Supplementary Materials

The following are available online at https://www.mdpi.com/article/10.3390/diagnostics11060917/s1, Table S1: Peptides; Table S2: Protein cargo of plasma-derived EVs from three patients with non-relapsed pediatric HL (explorative group), identified by shotgun LC-MS/MS; Table S3: Plasma EV proteins, Table S4: DAVID_RawData.
