# Supplementary material for: Proteomic Exploration of Plasma Exosomes and Other Small Extracellular Vesicles in Pediatric Hodgkin Lymphoma: A Potential Source of Biomarkers for Relapse Occurrence
Source: Diagnostics (Basel). 2021 May 21;11(6):917. doi: 10.3390/diagnostics11060917 (PMC8223799; doi:10.3390/diagnostics11060917)
Supplement: Supplementary file 1 [file diagnostics-11-00917-s001.zip › supplementary-revised-ok/Supplementary Table S2.doc]

**Table S2.** Protein cargo of plasma-derived EVs from three patients with non-relapsed pediatric HL (explorative group), identified by shotgun LC-MS/MS.

| **UniProt entry** | **Gene** | **Protein** | **Score** | **Known Presence in Exosomes of Human Sample Materials (ExoCarta Database)** | **[58]** | **[59]** | **[60]** |
| --- | --- | --- | --- | --- | --- | --- | --- |
| P00734 | F2 | Prothrombin | 323.31 | colorectal cancer cells, melanoma cells, platelets, prostate cancer cells | - | ✓ | - |
| P01009 | SERPINA1 | α-1-antitrypsin (or SERPINA1)* | 323.31 | colorectal cancer cells, hepatocellular carcinoma cells, hepatocytes, mesenchymal stem cells, plasma, platelets, thymus, urine | ✓ | - | ✓ |
| P01023 | A2M | α-2-macroglobulin* | 323.31 | B cells, colorectal cancer cells, endothelial cells, hepatocellular carcinoma cells, hepatocytes, keratinocytes, liver cancer cells, melanoma cells, mesenchymal stem cells, neuroblastoma cells, ovarian cancer cells, plasma, platelets, prostate cancer cells, synovial fluid, thymus | ✓ | ✓ | ✓ |
| P01024 | C3 | Complement C3* | 323.31 | hepatocellular carcinoma cells, hepatocytes, keratinocytes, melanoma cells, mesenchymal stem cells, nasopharyngeal carcinoma cells, ovarian cancer cells, plasma, platelets, prostate cancer cells, thymus, urine | ✓ | ✓ | ✓ |
| P02671 | FGA | Fibrinogen α chain* | 323.31 | hepatocytes, mesenchymal stem cells, plasma, platelets, thymus, urine | ✓ | ✓ | ✓ |
| P02675 | FGB | Fibrinogen β chain* | 323.31 | hepatocellular carcinoma cells, malignant pleural effusions, mesenchymal stem cells, plasma, platelets, synovial fluid, thymus | ✓ | ✓ | ✓ |
| P02679 | FGG | Fibrinogen γ chain°* | 323.31 | hepatocellular carcinoma cells, hepatocytes, malignant pleural effusions, mesenchymal stem cells, plasma, platelets, prostate cancer cells, synovial fluid, thymus, urine | ✓ | ✓ | ✓ |
| P02787 | TF | Serotransferrin | 323.31 | colorectal cancer cells, hepatocytes, keratinocytes, mesenchymal stem cells, nasopharyngeal carcinoma cells, platelets, prostate cancer cells, thymus, urine | ✓ | ✓ | - |
| P0C0L5 | C4B | Complement C4-B°* | 323.31 | melanoma cells, ovarian cancer cells, plasma, platelets, prostate cancer cells, saliva, thymus, urine | ✓ | ✓ | ✓ |
| P12259 | F5 | Coagulation factor V | 323.31 | colorectal cancer cells, hepatocellular carcinoma cells, hepatocytes, melanoma cells, platelets, prostate cancer cells | - | - | - |
| P19823 | ITIH2 | Inter-α-trypsin inhibitor heavy chain H2° | 323.31 | B cells, melanoma cells, mesenchymal stem cells, ovarian cancer cells, platelets, prostate cancer cells, | - | ✓ | - |
| P19827 | ITIH1 | Inter-α-trypsin inhibitor heavy chain H1 | 323.31 | melanoma cells, platelets, prostate cancer cells | - | ✓ | - |
| Q06033 | ITIH3 | Inter-α-trypsin inhibitor heavy chain H3 | 323.31 | melanoma cells, platelets, prostate cancer cells | - | - | - |
| Q14520 | HABP2 | Hyaluronan-binding protein 2 | 323.31 | unknown | - | - | - |
| P49747 | COMP | Cartilage oligomeric matrix protein | 313.47 | mesenchymal stem cells, prostate cancer cells | - | - | - |
| P00738 | HP | Haptoglobin°* | 305.75 | colorectal cancer cells, hepatocellular carcinoma cells, mesenchymal stem cells, plasma, platelets, thymus, urine | ✓ | ✓ | ✓ |
| P01871 | IGHM | Immunoglobulin heavy constant region mu° | 296.35 | breast milk, colorectal cancer cells, malignant pleural effusions, mesenchymal stem cells, platelets, saliva | - | - | - |
| P00740 | F9 | Coagulation factor IX | 280.21 | prostate cancer cells | - | - | - |
| P04003 | C4BPA | C4b-binding protein α chain* | 267.42 | plasma, platelets | ✓ | ✓ | ✓ |
| P00742 | F10 | Coagulation factor X | 266.52 | hepatocellular carcinoma cells, melanoma cells, prostate cancer cells | - | - | - |
| P10909 | CLU | Clusterin°* | 262.80 | breast milk, colorectal cancer cells, keratinocytes, mesenchymal stem cells, ovarian cancer cells, plasma, platelets, prostate cancer cells, saliva, squamous carcinoma cells, synovial fluid, thymus, urine | ✓ | ✓ | ✓ |
| P02647 | APOA1 | Apolipoprotein A-I° | 260.42 | colorectal cancer cells, hepatocellular carcinoma cells, hepatocytes, liver cancer cells, melanoma cells, mesenchymal stem cells, platelets, prostate cancer cells, saliva, thymus, urine | ✓ | ✓ | - |
| P04114 | APOB | Apolipoprotein B-100 | 254.47 | unknown | ✓ | - | ✓ |
| P0DOX7 | N/A | Immunoglobulin kappa light chain | 242.93 | unknown | - | - | - |
| P02760 | AMBP | Protein AMBP | 231.82 | hepatocellular carcinoma cells, urine | - | ✓ | - |
| P01857 | IGHG1 | Immunoglobulin heavy constant γ 1 | 221.49 | colorectal cancer cells, malignant pleural effusions, mesenchymal stem cells, platelets | - | - | - |
| P35443 | THBS4 | Thrombospondin-4 | 218.14 | prostate cancer cells | - | - | - |
| Q08380 | LGALS3BP | Galectin-3-binding protein* | 216.35 | bladder cancer cells, colorectal cancer cells, hepatocellular carcinoma cells, hepatocytes, keratinocytes, melanoma cells, mesenchymal stem cells, ovarian cancer cells, plasma, platelets, prostate cancer cells, saliva, squamous carcinoma cells, thymus, urine | ✓ | ✓ | ✓ |
| Q13201 | MMRN1 | Multimerin-1* | 202.89 | plasma, platelets | - | - | ✓ |
| P02774 | GC | Vitamin D-binding protein | 170.81 | colorectal cancer cells, melanoma cells, prostate cancer cells | - | ✓ | - |
| P51884 | LUM | Lumican | 170.61 | melanoma cells, prostate cancer cells | - | - | - |
| P00450 | CP | Ceruloplasmin | 165.81 | colorectal cancer cells, hepatocellular carcinoma cells, ovarian cancer cells, platelets, thymus, urine | ✓ | ✓ | - |
| P01011 | SERPINA3 | α-1-antichymotrypsin | 153.68 | melanoma cells, platelets, thymus, urine | - | ✓ | - |
| P04004 | VTN | Vitronectin* | 153.39 | colorectal cancer cells, hepatocellular carcinoma cells, melanoma cells, mesenchymal stem cells, plasma, platelets, prostate cancer cells, saliva, squamous carcinoma cells, thymus, urine | - | ✓ | ✓ |
| P01876 | IGHA1 | Immunoglobulin heavy constant α 1 | 152.71 | B cells, breast milk, mesenchymal stem cells, platelets, saliva, urine | - | - | - |
| P02649 | APOE | Apolipoprotein E* | 149.89 | B cells, breast milk, hepatocellular carcinoma, hepatocytes, keratinocytes, melanoma cells, mesenchymal stem cells, ovarian cancer cells, plasma, platelets, prostate cancer cells, thymus, urine | ✓ | - | ✓ |
| P68871 | HBB | Hemoglobin subunit β* | 140.18 | hepatocellular carcinoma cells, hepatocytes, liver cancer cells, mesenchymal stem cells, plasma, platelets, prostate cancer cells, thymus, urine | ✓ | ✓ | ✓ |
| P22891 | PROZ | Vitamin K-dependent protein Z | 135.67 | urine | - | - | - |
| B9A064 | IGLL5 | Immunoglobulin λ-like polypeptide 5 | 132.79 | platelets | ✓ | - | - |
| P02790 | HPX | Hemopexin | 131.11 | colorectal cancer cells, hepatocellular carcinoma cells, mesenchymal stem cells, nasopharyngeal carcinoma cells, neuroblastoma cells, prostate cancer cells, thymus, urine | - | ✓ | - |
| Q14624 | ITIH4 | Inter-α-trypsin inhibitor heavy chain H4 | 119.85 | melanoma cells, mesenchymal stem cells, nasopharyngeal carcinoma cells, platelets, prostate cancer cells, urine | - | ✓ | - |
| Q14515 | SPARCL1 | SPARC-like protein 1 | 116.77 | unknown | - | - | - |
| P05546 | SERPIND1 | Heparin cofactor 2 | 111.45 | platelets, prostate cancer cells | - | - | - |
| P55072 | VCP | Transitional endoplasmic reticulum ATPase | 109.90 | B cells, bladder cancer cells, colorectal cancer cells, keratinocytes, liver cancer cells, melanoma cells, mesenchymal stem cells, neuroblastoma cells, ovarian cancer cells, platelets, prostate cancer cells, thymus, urine | ✓ | - | - |
| P13591 | NCAM1 | Neural cell adhesion molecule 1 | 108.42 | melanoma cells, neuroblastoma cells, ovarian cancer cells | - | - | - |
| P01860 | IGHG3 | Immunoglobulin heavy constant γ 3 | 106.87 | colorectal cancer cells, platelets | - | - | - |
| P02751 | FN1 | Fibronectin* | 103.73 | bladder cancer cells, colorectal cancer cells, hepatocytes, keratinocytes, melanoma cells, mesenchymal stem cells, mesothelioma cells, neuroblastoma cells, ovarian cancer cells, plasma, platelets, prostate cancer cells, squamous carcinoma cells, synovial fluid, thymus, trophoblast cells, urine | ✓ | ✓ | ✓ |
| P24821 | TNC | Tenascin | 103.32 | hepatocellular carcinoma cells, keratinocytes, melanoma cells, mesenchymal stem cells, prostate cancer cells, squamous carcinoma cells | - | - | - |
| P05155 | SERPING1 | Plasma protease C1 inhibitor | 92.94 | unknown | - | ✓ | ✓ |
| P14625 | HSP90B1 | Endoplasmin | 92.58 | B cells, bladder cancer cells, colorectal cancer cells, keratinocytes, liver cancer cells, melanoma cells, mesenchymal stem cells, neuroblastoma cells, ovarian cancer cells, platelets, prostate cancer cells, saliva, thymus | - | - | - |
| P02765 | AHSG | α-2-HS-glycoprotein | 86.31 | B cells, colorectal cancer cells, hepatocellular carcinoma, hepatocytes, melanoma cells, mesenchymal stem cells, neuroblastoma cells, platelets, prostate cancer cells, urine | ✓ | ✓ | - |
| P07359 | GP1BA | Platelet glycoprotein Ib α chain | 86.18 | platelets | - | - | - |
| P09486 | SPARC | SPARC | 83.78 | mesenchymal stem cells | - | - | - |
| P69905 | HBA1 | Hemoglobin subunit α* | 83.09 | B cells, bladder cancer cells, colorectal cancer cells, hepatocellular carcinoma, hepatocytes, ovarian cancer cells, plasma, platelets, urine | ✓ | ✓ | ✓ |
| P08603 | CFH | Complement factor H | 82.16 | malignant pleural effusions, urine | - | ✓ | - |
| P01042 | KNG1 | Kininogen-1 | 80.82 | platelets, prostate cancer cells, urine | ✓ | ✓ | - |
| P07900 | HSP90AA1 | Heat shock protein HSP 90-α | 80.81 | B cells, bladder cancer cells, colorectal cancer cells, hepatocellular carcinoma cells, intestinal epithelial cells, malignant pleural effusions, melanoma cells, mesenchymal stem cells, mesothelioma cells, nasopharyngeal carcinoma cells, neuroblastoma cells, ovarian cancer cells, platelets, prostate cancer cells, saliva, thymus, urine | - | - | - |
| P07225 | PROS1 | Vitamin K-dependent protein S* | 78.67 | plasma, platelets | ✓ | ✓ | ✓ |
| P00747 | PLG | Plasminogen | 74.60 | colorectal cancer cells, melanoma cells, platelets, prostate cancer cells, saliva | - | ✓ | - |
| P27169 | PON1 | Serum paraoxonase/arylesterase 1* | 74.35 | plasma, platelets | - | ✓ | ✓ |
| P02763 | ORM1 | α-1-acid glycoprotein 1° | 71.73 | urine | - | ✓ | - |
| P27797 | CALR | Calreticulin | 65.27 | bladder cancer cells, keratinocytes, melanoma cells, mesenchymal stem cells, ovarian cancer cells, prostate cancer cells, saliva, thymus, urine | - | - | - |
| P01019 | AGT | Angiotensinogen* | 65.24 | plasma, platelets | - | ✓ | ✓ |
| P00751 | CFB | Complement factor B | 62.18 | keratinocytes, mesenchymal stem cells | ✓ | ✓ | - |
| P02749 | APOH | β-2-glycoprotein 1 | 61.56 | hepatocellular carcinoma cells, prostate cancer cells, thymus | - | - | - |
| P14543 | NID1 | Nidogen-1 | 60.62 | melanoma cells, mesenchymal stem cells, nasopharyngeal carcinoma cells, urine | - | - | - |
| P01859 | IGHG2 | Immunoglobulin heavy constant γ 2° | 60.48 | colorectal cancer cells, mesenchymal stem cells, platelets | - | - | - |
| P06727 | APOA4 | Apolipoprotein A-IV° | 58.73 | platelets, urine | - | ✓ | - |
| P63104 | YWHAZ | 14-3-3 protein zeta/delta | 56.72 | B cells, bladder cancer cells, colorectal cancer cells, hepatocellular carcinoma cells, keratinocytes, melanoma cells, mesenchymal stem cells, neuroblastoma cells, ovarian cancer cells, platelets, prostate cancer cells, saliva, squamous carcinoma cells, thymus, tracheobronchial cells, urine | ✓ | ✓ | - |
| P05090 | APOD | Apolipoprotein D* | 53.51 | plasma, platelets, urine | - | - | ✓ |
| P02748 | C9 | Complement component C9 | 53.33 | colorectal cancer cells, melanoma cells, ovarian cancer cells, platelets, prostate cancer cells, urine | - | ✓ | - |
| A1L4H1 | SSC5D | Soluble scavenger receptor cysteine-rich domain-containing protein SSC5D | 50.15 | unknown | - | - | - |
| P01861 | IGHG4 | Immunoglobulin heavy constant γ 4 | 48.83 | colorectal cancer cells, mesenchymal stem cells, platelets | - | - | - |
| P18428 | LBP | Lipopolysaccharide-binding protein | 47.70 | platelets, urine | - | - | - |
| P0DJI8 | SAA1 | Serum amyloid A-1 protein | 45.30 | prostate cancer cells, urine | - | - | - |
| P05067 | APP | Amyloid-β A4 protein | 37.42 | colorectal cancer cells, hepatocellular carcinoma cells, keratinocytes, mesenchymal stem cells, neuroblastoma cells, ovarian cancer cells, prostate cancer cells, saliva | - | - | - |
| P01008 | SERPINC1 | Antithrombin-III | 37.03 | unknown | - | ✓ | - |
| P08514 | ITGA2B | Integrin α-IIb | 35.75 | B cells, colorectal cancer cells, hepatocellular carcinoma cells, hepatocytes, melanoma cells, mesenchymal stem cells, neuroblastoma cells, ovarian cancer cells, platelets, prostate cancer cells | ✓ | ✓ | ✓ |
| O14791 | APOL1 | Apolipoprotein L1* | 35.20 | plasma, platelets | ✓ | ✓ | ✓ |
| P14151 | SELL | L-selectin | 34.84 | platelets | - | - | - |
| P63261 | ACTG1 | Actin, cytoplasmic 2 | 33.61 | breast cancer cells, colorectal cancer cells, hepatocellular carcinoma cells, hepatocytes, keratinocytes, melanoma cells, neuroblastoma cells, platelets, prostate cancer cells, squamous carcinoma cells, urine | ✓ | ✓ | - |
| P08697 | SERPINF2 | α-2-antiplasmin | 32.41 | melanoma cells, prostate cancer cells | - | - | - |
| P04217 | A1BG | α-1B-glycoprotein | 31.99 | hepatocellular carcinoma cells, prostate cancer cells, urine | ✓ | ✓ | - |
| P13727 | PRG2 | Bone marrow proteoglycan | 30.16 | unknown | - | - | - |
| P00739 | HPR | Haptoglobin-related protein* | 28.72 | colorectal cancer cells, mesenchymal stem cells, plasma, platelets, urine | ✓ | ✓ | ✓ |
| O95810 | CAVIN2 | Caveolae-associated protein 2 | 28.11 | unknown | - | - | - |
| P14314 | PRKCSH | Glucosidase 2 subunit β | 27.12 | colorectal cancer cells, ovarian cancer cells, prostate cancer cells | - | - | - |
| P05106 | ITGB3 | Integrin β-3 | 27.03 | B cells, colorectal cancer cells, hepatocellular carcinoma cells, hepatocytes, melanoma cells, neuroblastoma cells, ovarian cancer cells, platelets, prostate cancer cells | ✓ | - | - |
| Q99983 | OMD | Osteomodulin | 26.18 | unknown | - | - | - |
| P04406 | GAPDH | Glyceraldehyde-3-phosphate dehydrogenase | 24.53 | aqueous humor, B cells, bladder cancer cells, colorectal cancer cells, hepatocellular carcinoma cells, hepatocytes, intestinal epithelial cells, keratinocytes, melanoma cells, mesenchymal stem cells, mesothelioma cells, nasopharyngeal carcinoma cells, neuroblastoma cells, ovarian cancer cells, platelets, prostate cancer cells, saliva, squamous carcinoma cells, trabecular meshwork cells, tracheobronchial cells, urine | ✓ | - | - |
| P67936 | TPM4 | Tropomyosin α-4 chain | 23.96 | B cells, colorectal cancer cells, endothelial cells, ovarian cancer cells, platelets, prostate cancer cells | ✓ | - | - |
| P02766 | TTR | Transthyretin°* | 23.79 | hepatocellular carcinoma cells, plasma, platelets | ✓ | - | ✓ |
| P01031 | C5 | Complement C5 | 23.52 | colorectal cancer cells, melanoma cells, platelets, prostate cancer cells, urine | - | - | - |
| P10645 | CHGA | Chromogranin-A | 22.38 | unknown | - | - | - |
| P27105 | STOM | Erythrocyte band 7 integral membrane protein | 22.05 | breast milk, hepatocellular carcinoma cells, hepatocytes, melanoma cells, mesenchymal stem cells, neuroblastoma cells, ovarian cancer cells, platelets, prostate cancer cells, thymus, tracheobronchial cells, urine | ✓ | ✓ | - |
| P04275 | VWF | von Willebrand factor* | 21.12 | hepatocytes, plasma, platelets, thymus, urine | ✓ | - | ✓ |
| P55209 | NAP1L1 | Nucleosome assembly protein 1-like 1 | 20.64 | colorectal cancer cells, melanoma cells, neuroblastoma cells, ovarian cancer cells, platelets, prostate cancer cells, squamous carcinoma cells, thymus | - | - | - |
| P20160 | AZU1 | Azurocidin | 20.13 | urine | - | - | - |
| P08185 | SERPINA6 | Corticosteroid-binding globulin | 19.67 | unknown | - | - | - |
| P04196 | HRG | Histidine-rich glycoprotein | 19.50 | platelets, urine | - | ✓ | - |
| P07237 | P4HB | Protein disulfide-isomerase | 19.19 | bladder cancer cells, colorectal cancer cells, hepatocellular carcinoma cells, melanoma cells, mesenchymal stem cells, ovarian cancer cells, platelets, prostate cancer cells, saliva, thymus, urine | - | - | - |
| P25311 | AZGP1 | Zinc-α-2-glycoprotein | 18.76 | liver cancer cells, platelets, saliva, serum, urine | - | - | - |
| P01619 | IGKV3-20 | Immunoglobulin kappa variable 3-20 | 18.59 | colorectal cancer cells, saliva | - | - | - |
| O43866 | CD5L | CD5 antigen-like* | 18.58 | colorectal cancer cells, plasma, platelets, synovial fluid | ✓ | ✓ | ✓ |
| Q9Y490 | TLN1 | Talin-1 | 18.39 | B cells, colorectal cancer cells, hepatocellular carcinoma cells, keratinocytes, melanoma cells, mesenchymal stem cells, nasopharyngeal carcinoma cells, neuroblastoma cells, ovarian cancer cells, platelets, prostate cancer cells, squamous carcinoma cells, thymus, urine | ✓ | - | - |
| Q14766 | LTBP1 | Latent-transforming growth factor β-binding protein 1 | 18.21 | colorectal cancer cells, mesenchymal stem cells, platelets | - | - | - |
| P61981 | YWHAG | 14-3-3 protein gamma | 16.77 | B cells, bladder cancer cells, colorectal cancer cells, endothelial cells, hepatocellular carcinoma cells, keratinocytes, melanoma cells, mesenchymal stem cells, neuroblastoma cells, ovarian cancer cells, platelets, prostate cancer cells, saliva, thymus, urine | - | - | - |
| P04070 | PROC | Vitamin K-dependent protein C | 15.99 | unknown | - | - | - |
| P68366 | TUBA4A | Tubulin α-4A chain | 15.06 | B cells, bladder cancer cells, colorectal cancer cells, keratinocytes, neuroblastoma cells, ovarian cancer cells, platelets, prostate cancer cells, saliva, squamous carcinoma cells, thymus, urine | - | - | - |
| P16112 | ACAN | Aggrecan core protein | 14.83 | unknown | - | - | - |
| P43652 | AFM | Afamin | 14.64 | hepatocellular carcinoma cells, mesenchymal stem cells | - | ✓ | - |
| P19652 | ORM2 | α-1-acid glycoprotein 2 | 14.59 | unknown | - | ✓ | - |
| P07357 | C8A | Complement component C8 α chain | 14.56 | platelets | - | ✓ | ✓ |
| P09871 | C1S | Complement C1s subcomponent | 14.39 | mesenchymal stem cells, platelets | ✓ | ✓ | - |
| P01834 | IGKC | Immunoglobulin kappa constant | 14.35 | breast milk, colorectal cancer cells, mesenchymal stem cells, platelets, saliva | - | - | - |
| P08519 | LPA | Apolipoprotein(a)* | 14.32 | plasma, platelets | - | - | ✓ |
| A0A0B4J1Y9 | IGHV3-72 | Immunoglobulin heavy variable 3-72 | 14.21 | unknown | - | - | - |
| P0DOX2 | N/A | Immunoglobulin α-2 heavy chain | 13.77 | unknown | - | - | - |
| P01717 | IGLV3-25 | Immunoglobulin λ variable 3-25 | 13.70 | unknown | - | - | - |
| P31946 | YWHAB | 14-3-3 protein β/α | 13.67 | B cells, bladder cancer cells, colorectal cancer cells, keratinocytes, melanoma cells, mesenchymal stem cells, neuroblastoma cells, ovarian cancer cells, platelets, prostate cancer cells, saliva, thymus, urine | - | - | - |
| P13671 | C6 | Complement component C6 | 13.39 | platelets | ✓ | - | - |
| P61224 | RAP1B | Ras-related protein Rap-1b | 13.35 | B cells, colorectal cancer cells, hepatocellular carcinoma cells, hepatocytes, melanoma cells, mesenchymal stem cells, neuroblastoma cells, ovarian cancer cells, platelets, prostate cancer cells, saliva, squamous carcinoma cells, thymus, urine | - | - | - |
| P16070 | CD44 | CD44 antigen | 13.30 | B cells, bladder cancer cells, colorectal cancer cells, endothelial cells, hepatocellular carcinoma cells, keratinocytes, melanoma cells, mesenchymal stem cells, nasopharyngeal carcinoma cells, ovarian cancer cells, platelets, prostate cancer cells, saliva, thymus, urine | - | - | - |
| Q7Z7G0 | ABI3BP | Target of Nesh-SH3 | 13.20 | mesenchymal stem cells | - | - | - |
| P02776 | PF4 | Platelet factor 4 | 13.02 | unknown | - | - | - |
| P11597 | CETP | Cholesteryl ester transfer protein | 12.95 | platelets, urine | - | - | - |
| P0C0L4 | C4A | Complement C4-A°* | 12.85 | malignant pleural effusions, melanoma cells, ovarian cancer cells, plasma, platelets, prostate cancer cells, thymus, urine | ✓ | ✓ | ✓ |
| P02741 | CRP | C-reactive protein | 12.83 | unknown | - | - | - |
| P62258 | YWHAE | 14-3-3 protein ε | 12.42 | B cells, bladder cancer cells, colorectal cancer cells, keratinocytes, melanoma cells, mesenchymal stem cells, neuroblastoma cells, ovarian cancer cells, platelets, prostate cancer cells, saliva, squamous carcinoma cells, thymus, urine | - | - | - |
| P20851 | C4BPB | C4b-binding protein β chain* | 12.40 | plasma | - | - | - |
| P08311 | CTSG | Cathepsin G | 12.40 | mesenchymal stem cells, thymus, urine | - | - | - |
| P0DOY2 | IGLC2 | Immunoglobulin λ constant 2 | 11.91 | breast milk, mesenchymal stem cells, platelets | - | - | - |
| P13611 | VCAN | Versican core protein | 11.55 | melanoma cells, mesenchymal stem cells | - | - | - |
| P21333 | FLNA | Filamin-A | 11.53 | B cells, bladder cancer cells, colorectal cancer cells, hepatocellular carcinoma cells, hepatocytes, keratinocytes, liver cancer cells, melanoma cells, mesenchymal stem cells, nasopharyngeal carcinoma cells, neuroblastoma cells, ovarian cancer cells, platelets, prostate cancer cells, thymus, urine | ✓ | - | - |
| Q96NW7 | LRRC7 | Leucine-rich repeat-containing protein 7 | 11.09 | unknown | - | - | - |
| Q9Y3X0 | CCDC9 | Coiled-coil domain-containing protein 9 | 10.98 | unknown | - | - | - |
| P54278 | PMS2 | Mismatch repair endonuclease PMS2 | 10.95 | unknown | - | - | - |
| P02656 | APOC3 | Apolipoprotein C-III | 10.85 | colorectal cancer cells, liver cancer cells, melanoma cells, platelets, prostate cancer cells | - | - | - |
| P23083 | IGHV1-2 | Immunoglobulin heavy variable 1-2 | 10.81 | unknown | - | - | - |
| Q5T9S5 | CCDC18 | Coiled-coil domain-containing protein 18 | 10.71 | unknown | - | - | - |
| P0DP03 | IGHV3-30-5 | Immunoglobulin heavy variable 3-30-5 | 10.02 | unknown | - | - | - |
| P02730 | SLC4A1 | Band 3 anion transport protein | 9.28 | platelets, urine | ✓ | ✓ | - |
| A0A075B6K5 | IGLV3-9 | Immunoglobulin λ variable 3-9 | 9.18 | unknown | - | - | - |
| P10124 | SRGN | Serglycin | 8.88 | mesenchymal stem cells, prostate cancer cells | - | - | - |
| P08238 | HSP90AB1 | Heat shock protein HSP 90-β | 8.86 | B cells, bladder cancer cells, colorectal cancer cells, hepatocellular carcinoma cells, melanoma cells, mesenchymal stem cells, mesothelioma cells, ovarian cancer cells, platelets, prostate cancer cells, thymus, urine | - | - | - |
| P01591 | JCHAIN | Immunoglobulin J chain* | 8.31 | mesenchymal stem cells, plasma, saliva, thymus, urine | - | - | ✓ |
| P06310 | IGKV2-30 | Immunoglobulin kappa variable 2-30 | 7.72 | unknown | - | - | - |
| P30512 | HLA-A | HLA class I histocompatibility antigen, A-29 α chain | 7.56 | B cells, bladder cancer cells, breast milk, colorectal cancer cells, hepatocellular carcinoma cells, hepatocytes, intestinal epithelial cells, keratinocytes, melanoma cells, neuroblastoma cells, ovarian cancer cells, platelets, saliva, thymus, urine | ✓ | - | - |
| Q99733 | NAP1L4 | Nucleosome assembly protein 1-like 4 | 7.23 | B cells, hepatocellular carcinoma cells, hepatocytes, neuroblastoma cells, thymus | - | - | - |
| P07358 | C8B | Complement component C8 β chain | 6.95 | unknown | - | - | - |
| Q96L50 | LRR1 | Leucine-rich repeat protein 1 | 6.89 | unknown | - | - | - |
| P01714 | IGLV3-19 | Immunoglobulin λ variable 3-19 | 6.53 | unknown | - | - | - |
| A0A0B4J1U7 | IGHV6-1 | Immunoglobulin heavy variable 6-1 | 6.47 | unknown | - | - | - |
| P55058 | PLTP | Phospholipid transfer protein | 6.45 | keratinocytes, mesenchymal stem cells, platelets, prostate cancer cells, saliva, squamous carcinoma cells | - | - | - |
| P02750 | LRG1 | Leucine-rich α-2-glycoprotein | 6.37 | urine | - | - | - |
| Q9UI32 | GLS2 | Glutaminase liver isoform, mitochondrial | 6.34 | unknown | - | - | - |
| Q92954 | PRG4 | Proteoglycan 4* | 6.33 | plasma | - | - | ✓ |
| P13667 | PDIA4 | Protein disulfide-isomerase A4 | 6.33 | bladder cancer cells, colorectal cancer cells, endothelial cells, melanoma cells, nasopharyngeal carcinoma cells, ovarian cancer cells, squamous carcinoma cells, thymus | - | - | - |
| P48788 | TNNI2 | Troponin I, fast skeletal muscle | 6.33 | urine | - | - | - |
| P10321 | HLA-C | HLA class I histocompatibility antigen, Cw-7 α chain | 6.29 | B cells, bladder cancer cells, colorectal cancer cells, malignant pleural effusions, melanoma cells, nasopharyngeal carcinoma cells, neuroblastoma cells, ovarian cancer cells, squamous carcinoma cells, urine | - | - | - |
| Q9Y5P2 | CSAG2 | Chondrosarcoma-associated gene 2/3 protein | 6.27 | unknown | - | - | - |

° Identified by DIGE; * Previously identified in exosomes from human plasma (www.exocarta.org).
